# The Endosphere Microbiome of Ginseng

**DOI:** 10.3390/plants11030415

**Published:** 2022-02-02

**Authors:** Paul H. Goodwin

**Affiliations:** School of Environmental Sciences, University of Guelph, 50 Stone Road East, Guelph, ON N1G 2W1, Canada; pgoodwin@uoguelph.ca

**Keywords:** archaea, bacteria, endophyte, fungi, Panax, stress, symbioses

## Abstract

The endosphere of ginseng contains a variety of fungal, bacterial, archaeal and viral endophytes. Bacterial endophytes are primarily members of the Proteobacteria, Actinobacteria, Firmicutes and Bacteroidetes, and fungal endophytes are primarily members of the Ascomycota, Zygomycota and Basidiomycota. Although archaea and viruses have been detected in symptomless ginseng plants, little is known about them. Many but not all studies have shown roots having the highest abundance and diversity of bacterial and fungal endophytes, with some endophytes showing specificity to above or belowground tissues. Abundance often increases with root age, although diversity can decrease, possibly related to increases in potential latent fungal pathogen infections. The descriptions of many endophytes that can metabolize ginsenosides indicate an adaptation of the microbes to the unique combination of secondary metabolites found in ginseng tissues. Most research on the benefits provided by bacterial and fungal endophytes has concentrated on improved plant nutrition, growth promotion and increased disease resistance, but little on their ability to increase abiotic stress resistance. Some other areas where more research is needed is field trials with endophyte-treated plants grown in various environments, genomic/metagenomic analysis of endophytes, and the effects of endophytes on induced disease resistance and abiotic stress tolerance.

## 1. Introduction

An endophyte has been defined as a fungus or bacterium colonizing the internal tissues of a plant without resulting in visible disease symptoms, which would include microbes that are beneficial as well as having no apparent effect on the plant [1]. The internal tissues of plants are termed the endosphere [2]. Bacterial endophytes can infect roots through wounds or direct penetration growing biotrophically in the apoplast and then invade stems by passing through the vascular system or apoplast of the stem spreading to leaves, flowers and seeds. They can also infect other tissues directly, such as entering the leaf surface via wounds, natural openings or direct penetration [2,3]. Insects can also act as endophyte vectors when feeding on sap and pollinating [4].

For most plants, bacterial endophytes are primarily in the γ-, α-, β-Proteobacteria, Firmicutes and Actinobacteria [5]. The diversity of bacterial endophytes can be affected by many factors, including plant tissue type, plant development, soil type and environmental conditions. Diversity and abundance are usually highest in roots, followed by stems and then leaves for woody plants, but there is considerable variation among herbaceous angiosperms [6]. Diversity typically increases with age during a growing season and over years for perennial plants [7]. Soils higher in organic matter tend to favor endophytes, while unfavorable environmental conditions for plants tend to reduce bacterial endophyte abundance and diversity [8]. 

Fungal endophytes in most plants are primarily members of the Ascomycota, Basidiomycota and Zygomycota [9]. Their diversity is affected by similar factors as bacterial endophytes but with some differences, such as often having higher diversity and abundance in roots but then followed by leaves and then stems [10]. However, there is also considerable variation between different plants and different fungal endophytes. Their abundance and diversity are generally affected by environmental and plant developmental factors similar to that of bacterial endophytes. 

Archaeal endophytes are much less studied but have been found by DNA-based detection, such as 16S rRNA sequencing, and mostly belong to the Cenarchaea and Euryarchaea [11]. In some cases, a second round of semi-nested PCR with an archaea-specific primer is needed to detect them, possibly indicating very low populations inside plants [12]. With so few studies on archaeal endophytes, little is known about how they are affected by plant genotype, development and stress. However, stems and leaves were colonized by soil archaea that had entered roots, and so it is likely that soil conditions would impact endophytic archaea like they do for endophytic bacteria [12]. 

The existence of viral endophytes is controversial. However, some plant viruses do not cause noticeable symptoms in plants, like bacteria and fungi, and thus could be considered endophytic based on the definition of Wilson [1]. Such viruses are often termed cryptic or persistent [13] or much more rarely termed endophytic [14]. The term cryptic is used because there are no symptoms in plants, while persistent is used because they have a long-term dependent association with their host. Persistent viruses have been identified from apparently healthy plants using high-throughput sequencing of plant RNA or DNA, and are primarily members of the Endornavirues, Partitiviruses, Chrysoviruses and Totivirueses based on sequence comparisons [15]. Thus far, there is limited information about them, and it is not known if they are affected by factors such as plant development and stress.

Ginseng (*Panax*) is a genus of herbaceous perennial plants in the Araliaceae that are both harvested from the wild and cultivated commercially and are used as a medicinal herb primarily due to secondary metabolites in the roots, particularly a variety of ginsenosides (teriterpenoid saponins) [16]. There are 16 *Panax* species native to central and eastern Asia or eastern North America [17], but the primary cultivated species are American ginseng (*Panax quinquefolius*) and Asian ginseng (*Panax ginseng*), and to a lesser extent Himalayan ginseng (*Panax notoginseng*). Cultivation of ginseng is a relatively lengthy and costly process, requiring considerable inputs [18]. Many of the studies of ginseng endophytes have been prompted by trying to find ways to either reduce those inputs, such as using endophytes to help control plant diseases instead of pesticides, improve plant nutrition to reduce chemical fertilization, promote plant growth, increase root ginsenoside content and transform common root ginsenosides into rarer more biologically active forms. This review will concentrate on the factors affecting ginseng endophyte diversity and abundance, as well as benefits to ginseng provided by the endophytes. 

## 2. Ginseng Bacterial Endophyte Diversity and Abundance

A range of bacterial endophytes in two *Panax* species have been identified by isolation or direct DNA sequencing from plant tissues. In *P. ginseng*, direct 16S rDNA sequencing revealed that the bacterial endophytes in 2- to 6-year-old roots were mostly Proteobacteria (50–70%) followed by Actinobacteria (20–40%), with much fewer Firmicutes and Bacteroidetes and then very small numbers belonging to the Chlamydiae, Chloroflexi, Fusobacteria, Spirochaetes and Tenericutes [19]. Using isolation, Chowdury et al. [20] also found that endophytes in *P. ginseng* roots, stems and leaves were mostly Proteobacteria (63%), and the next most common were the Firmicutes (30%), followed by the Bacteroidetes (4%) and Actinobacteria (2%). In contrast, Vendan et al. [21], also using isolation, detected predominantly Firmicutes (86%) in *P. ginseng* stems, but there were also Proteobacteria (10%) and Actinobacteria (4%). For *P. notoginseng,* direct 16S rDNA sequencing was used to show that the primary bacterial phyla in leaves, stems, fruits and flowers were Proteobacteria, Actinobacteria, Verrumicrobia, Bacteroidetes, and Acidobacteria with smaller numbers in the Firmicutes, Gemmatimonadetes, Chloroflexi, Planctomycetes and Cyanobacteria [22]. The most abundant were Proteobacteria, ranging from 33 to 62% in abundance between tissues, and the next most abundant was Actinobacteria, ranging from 13 to 22%. For *P. quinquefolius,* direct 16S rDNA sequencing showed that leaves, stems and roots also had Proteobacteria, Actinobacteria, Bacteroidetes, Plantomycetes and Acidobacteria as the most abundant phyla, with Proteobacteria being the most abundant in all tissues, ranging from 41 to 73% [23]. By comparison, the Bacteroidetes ranged from at 7 to 18% and Actinobacteria ranged from 6 to 8% between different tissues. Comparing the studies, it appears that the most abundant was always the Proteobacteria at one- to two-thirds abundance in the three *Panax* species, except for Vendan et al. [21]. That could be due to Vendan et al. [21] only examining 51 isolates compared to 1,886 isolates by Chowdury et al. [20] and up to 10,351 operational taxonomic units (OTUs) by Hong et al. [19], Dong et al. [22] and Shehata et al. [23]. The Actinobacteria, Firmicutes and Bacteroidetes were typically the next most common phyla with considerable variation in abundance between studies. As well, there is a wide range of other bacterial phyla present at much lower abundances. 

Tissue type is an important factor determining bacterial endophyte diversity and abundance. For 21 bacterial endophytes isolated from *P. ginseng* seedlings, bacteria in leaves and rhizomes were more diverse than those in other tissues [24]. Based on direct 16S rDNA sequencing, endophytes in *P. ginseng* roots, stems, flower stalk and leaves belonged to the Actinobacteria, Firmicutes and Proteobacteria, but members of the Actinobacteria were not detected in flower stalks [25]. The highest abundance and diversity were in roots. While there were taxa specific to certain tissues, there were also common species in stems, roots, leaves, and flower stalks, such as *Bacillus amyloliquefaciens, Bacillus aryabhattai, Bacillus megaterium* and *Staphylococcus saprophyticus*. Another study of *P. ginseng* showed that culturable endophyte diversity was greatest in leaves followed by roots and lowest in stems, and stems and roots had the most similar bacterial endophytes suggesting that many endophytes first invaded the roots [21]. For *P. notoginseng*, direct 16S rDNA sequencing showed that Actinobacteria, Bacteroidetes, Verrucomicrobia, and Firmicutes were abundant in roots, Proteobacteria and Gemmatimonadetes were abundant in stems, and the Cytophagales were abundant in leaves [26]. There was greater diversity in roots compared to stems, which had greater diversity than leaves. Another study with direct 16S rDNA sequencing of *P. notoginseng* showed that bacterial endophytes were predominantly Proteobateria, Actinobacteria, Verrucomicrobia and Bacteroidetes in all tissues, but the endophytes in aboveground tissues (flower, leaf, and stem) were different from those in belowground tissues (root and fibril) [22]. At the genus level, *Conexibacter, Gemmatimonas, Holophaga, Luteolibacter, Methylophilus, Prosthecobacter* and *Solirubrobacter* were more abundant in aboveground than belowground tissues, whereas the reverse was true for *Bradyrhizobium, Novosphingobium, Phenylobacterium, Sphingobium* and *Steroidobacter*. For *P. quinquefolius*, direct 16S rDNA sequencing revealed that Proteobacteria were most abundant in all tissues, while Bacteroidetes and Actinobacteria were relatively abundant in leaves, Bacteroidetes, Acidobacteria and Actinobacteria relatively abundant in stems, and Plantomycetes, Bacteroidetes and Actinobacteria relatively abundant in roots [23]. At the genus level, *Acinetobacter* and *Sphingomonas* were most abundant in leaves, an unknown genus in Ellin6075, *Sphingomonas* and *Acinetobacter* in stems, and unidentified genera in the Pirellulaceae and Myxococcales in roots. Stems and roots had significantly higher diversity than leaves. While the studies differed, most often the highest abundance and diversity were observed the belowground tissues, with several differences in the endophyte genera between tissues. 

Plant age also affects ginseng bacterial endophyte diversity and abundance. In a study of 1886 bacterial endophytes isolated from *P. ginseng* seedlings, there were few differences before transplanting, but there were major changes correlated with transplantation, indicating an effect of the soil environment [24]. Endophytes in the Micrococcaceae, found only in leaves and rhizomes, were not detected prior to transplantation, and thus were presumed to have entered the tissues from the soil or air after transplantation. For 116 culturable bacterial endophytes from 2- to 6-year-old *P. ginseng*, the Firmicutes were abundant at all ages, except 3-year-old plants, which had the lowest diversity and the Proteobacteria were more abundant [25]. The greatest diversity was in 2-year-old plants. While some endophytes were specific to a plant age, other endophytes, such as *B. amyloliquefaciens* and *B. aryabhattai*, were found in plants of all ages. For 10 samples of 2- to 6-year-old *P. ginseng* plants with 16S rDNA sequencing, Hong et al. [19] showed that while members of the Proteobacteria and Actinobacteria were most abundant in all years, the Alphaproteobacteria were the most abundant in 3- to 5-year-old plants. Three-year-old plants had the highest endophyte abundance with some species, such as *Streptomyces cyaneusi* and *Dehlococcoides* sp., being unique to 3-year-old plants. The differences between the two studies may reflect using a culture-dependent technique with several tissue types versus a culture-independent technique with only roots. For *P. notoginseng*, bacterial endophytic diversity determined by 16S rDNA sequencing for 6 samples per age was highest in 3-year-old roots, followed by 1-year-old roots and then 2-year-old roots, and members of the Cyanobacteria, Proteobacteria, Actinobacteria, and Acidobacteria dominated for all the root ages [27]. Thus, it is difficult yet to conclude how bacterial endophytes are affected over the years of growth, but perhaps the overriding factors affecting the bacterial endophytes over time are differences in one or more environmental conditions, such as rainfall and temperature, between years. 

## 3. Ginseng Interactions with Bacterial Endophytes

One trait examined in bacterial endophytes isolated from ginseng is their ability to enzymatically convert ginsenosides in culture, which is typically performed to obtain ginsenosides with desirable pharmacological effects, such as Rg3 that is present in low amounts in ginseng roots [28]. For example, a β-glucosidase-producing endophytic bacterium was isolated from *P. ginseng* roots and identified a *Flavobacterium sp.* that could deglycosylate the ginsenoside Rb1 to the rare ginsenosides Gyp-XVII and Rg3 [28]. Another isolate of a β-glucosidase-producing endophytic bacterium from *P. ginseng* roots was a *Burkholderia* sp. that could hydrolyse glucosidic linkages at C-20 and C-3 of ginsenosides converting Rb1 to Rg3 [29]. A bacterial endophyte from roots of *P. ginseng* with ginsenoside conversion abilities is *Agrobacterium rhizogenes* that converted common ginsenosides into the rare ginsenosides F2, Rg3 and Rh2 [30]. In *P. notoginseng*, the bacterial endophytes *Brevundimonas* and *Bacillus* could convert Rg1 to Rh1 and Rg1 to vinaginsenoside possibly via glycosyltransferase and oxidase activities, respectively [31]. 

Ginsenosides can also be altered within ginseng tissues by bacterial endophyte inoculation. *Bacillus altitudinis* isolated from field-cultivated *P. ginseng* roots increased biomass and ginsenoside accumulation in ginseng adventitious root culture, likely by eliciting PAMP-triggered immunity (PTI) related to the defense hormone jasmonate, which also elicited ginsenoside accumulation [32]. Foliar inoculation of *P. ginseng* in the field with an isolate of *Paenibacillus polymyxa* from healthy leaves of *P. ginseng* increased plant growth and concentrations of Rg1, Re, Rf, Rb1, Rg2, Rb2, Rb3 and Rd [33]. Some ginsenosides were reduced with endophyte treatment compared to the control, such as Rc that was lowered by 54%, whereas others were greatly increased, such as Rd that was 308% higher than the control. Inoculation with *P. polymyxa* was believed to stimulate ginsenoside synthesis, probably by eliciting PTI. This would also be consistent with reduced plant mortality observed with *P. polymyxa* inoculation, possibly due to induced systemic resistance (ISR) that is associated with jasmonate-regulated defenses and often been reported to be triggered by endophytes [34]. A clear example of a bacterial root endophyte inducing ISR in ginseng is a soil drench with the bacterial endophyte, *B. amyloliquefaciens*, from *P. ginseng* roots that induced resistance against *Phytophthora cactorum* and upregulated the *P. ginseng* ISR defense-related markers genes, *PgPR5* (thaumatin-like)*, PgPR10* (ribonuclease) and *PgCAT* (ROS scavenging), in leaves following *P. cactorum* inoculation [35]. 

Increased disease resistance in ginseng can be due to the direct antimicrobial activities of endophytic bacteria. Isolation of 21 bacterial endophytes from different tissues of *P. ginseng* seedlings resulted in 10 isolates of *Paenibacillus, Bacillus* and *Pseudomonas* showing inhibition of both *Cylindrocarpon destructans* and *Botrytis cinerea* in culture with the clear inhibition zones, indicating antibiotic production [25]. Out of 116 isolates from various tissues of 2- to 6-year-old *P. ginseng* that belonged to 42 different species, *B. amyloliquefaciens, B. megaterium, Pseudomonas frederiksbergensis* and *Staphylococcus saprophyticus* inhibited *C. destructans* and *B. cinerea* in dual-cultures [24]. Isolation of 63 bacterial strains from 5-year-old *P. ginseng* roots resulted in *P. polymyxa*, a *Bacillus* species and *Pseudomonas poae* with inhibitory activity in culture against *Rhizoctonia solani, Fusarium oxysporum, Pythium ultimum* and *Phytophthora* *capsici* [36]. The authors noted that *P. polymyxa* and *Bacillus* are well-known antibiotic producers. An endophyte from *P. ginseng* roots, *Burkholderia stabilis,* produced antimicrobial compounds in culture that were fractionated by high-performance liquid chromatography and showed broad fungal inhibitory activity against *Pythium, Botrytis, Alternaria, Rhizoctonia* and *Cylindrocarpon*, although the putative antibiotics were not identified [37]. The endophyte reduced root rot symptoms caused by *C. destructans* when roots were dipped into the *B. stabilis* suspension, but the *B. stabilis*–treated roots infected with *C. destrucans* had lower expression of the defense genes, *PgPR2* (Beta-1,3-glucanase), *PgPR3* (chitinase), *PgPR4* (chitinase)*, PgPR5, PgPR6* (cysteine proteinase inhibitor-like), and *PgPR10* than *C. destructans*-infected roots without the endophyte, which indicated that ISR was not involved. For *P. notoginseng*, screening 1000 bacterial endophytes from roots, stems, petioles, leaves and seeds resulted in the identification of 104 isolates showing antimicrobial activity in culture against one of more of the root pathogens, *F. oxysporum* and *Ralstonia* sp. as well as nematicidal activity against *Meloidogyne hapla* [38]. Isolates with these activities were mostly from roots and were *Bacillus* species, which are well-known antibiotic producers, with *B. amyloliquefaciens* subsp. *plantarum*, *Bacillus methylotophicus, B. cereus* and *Bacillus sonorensis* having activity against all three parasites. Thus, there are many examples of bacterial endophytes directly inhibiting pathogen growth, most commonly isolates of *Paenibacillus, Bacillus* and *Pseudomonas*; however, only Kim et al. [37] demonstrated that a bacterial endophyte could also reduce the levels of disease. 

Bacterial endophytes can improve ginseng growth by increasing access to nutrients. Among 52 isolates from *P. ginseng* stems, isolates of *Strenotrophomonas maltophilia* and *Agrobacterium tumefaciens* contained *nifH*, indicating their capability to fix nitrogen [21]. Among those isolates, other mechanisms for improved nutrition were the ability to solubilize phosphate, which was found in half of the isolates with *Lysinibacillus fusiformis, B. cereus* and *B. megaterium* having the highest phosphate solubilization activities, but only seven isolates produced siderophores for possible iron scavenging. Among eight endophytes from *P. ginseng* seeds, two *Enterobacter* isolates showed the ability to solubilize phosphate and fix nitrogen [39]. An isolate of *P. fluorescens* from *P. ginseng* also contained *nifH*, indicating nitrogen fixation [40]. It also showed the ability to solubilize phosphate and produce siderophores. Among 252 bacterial endophytes from *P. ginseng* roots, stems and leaves, 85 were positive for siderophore production and 118 showed phosphate solubilization with both traits mostly found for isolates in the Proteobacteria, particularly for phosphate solubilization [20]. Prediction of bacterial functions from metagenomic data of bacterial endophytes in *P. ginseng* roots showed that genes were present for siderophore production, phosphate solubilization and nitrogen assimilation [19]. Thus, bacterial endophytes can enhance ginseng growth by making iron, phosphate and nitrogen more available, and quite frequently all three traits can be found in the same isolate. 

Bacterial endophytes can increase plant growth by affecting hormones in *Panax* species, such as indoleacetic acid (IAA) production that stimulates plant cell elongation. Among *P. ginseng* bacterial endophytes, 47 isolates produced IAA with *Micrococcus luteus, L. fusiformis* and *B. cereus* showing the highest IAA production [21]. Among 252 endophyte isolates, 32 showed IAA-like compound production primarily for members of the Proteobacteria and to a lesser extent for the Fimicutes and rarely for the Bacteriodetes and Actinobacteria [20]. Two *Enterobacter* endophytes from *P. ginseng* seeds had the ability to produce IAA [39]. 1-Aminocyclopropane-1-carboxylate (ACC) deaminase activity was found in an endophytic *P. fluorescens* from *P. ginseng*, indicating that it could stimulate growth by reducing stress-related ethylene that limits plant growth [40]. Analyzing metagenomic data from bacterial endophytes of *P. ginseng* also showed the presence of ACC deaminase genes [19]. It appears that many bacterial endophytes have the potential to promote ginseng growth through manipulation of plant hormones. 

## 4. Ginseng Fungal Endophyte Diversity and Abundance

Compared to bacterial endophytes, there have been fewer studies of fungal endophyte abundance and diversity in ginseng. Among fungal endophytes isolated from *P. ginseng*, the Ascomycota were 82%, Zygomycota were 10%, Basidiomycota were 7%, and unknowns were 1% of the isolates [41]. There were 59 genera, with *Trichoderma* being the most common at over 10%, and only the genera *Fusarium, Umbelopsis, Penicillium, Phomopsis, Phoma, Alternaria* and *Geomyces* occurring above 4% of the isolates, all of which belonged to the Ascomycota. Also using isolation from *P. ginseng*, all of the common genera, *Alternaria, Fusarium, Phoma, Penicillium* and *Entrophosphora*, belonged to Ascomycota [42]. Similarly, 97% and 3% of 89 fungal endophyte isolates obtained from *P. notoginseng* belonged to the Ascomycota and Zygomycota, respectively, and among the 23 genera, the most common were *Alternaria* and *Penicillium*, both belonging to the Ascomycota [43]. Using direct sequencing of the fungal nuclear ribosomal internal transcribed spacer1 (ITS1) with *P. notoginseng*, the Ascomycota were again dominant with 95% of the endophytes, with much fewer in the Zygomycota, Basidiomycota and Chytridiomycota, and the most common genera, *Emericella* (*Aspergillus*) and *Fusarium*, were both in the Ascomycota [44]. Using direct ITS2 sequencing, Wei et al. [26] also reported that endophytic fungi of *P. notoginseng* were dominated by Ascomycota (76%), with much fewer Basidiomycota (7%). Members of the Ascomycota were also predominant among 134 fungi isolated from *P. quinquefolius*, with all the isolates belonging to Ascomycota (*Glomerella* and *Cladosporium* being most common), except for the Basidiomycete *Scizophyllum* [45]. For both isolation and direct sequence analysis, all these studies showed a fungal endophyte abundance of three-quarters to almost all belonging in the Ascomycota, with much fewer numbers in the Zygomycota and Basidiomycota. 

There have been few studies on the diversity and abundance of fungal endophytes in different tissues of *Panax* species. For *P. notoginseng*, Zheng et al. [43] used culturing to find that roots had both the highest abundance and diversity, with isolates in nine genera, seeds had the second highest abundance but third highest diversity, stems had the third highest abundance but second highest diversity, and leaves had both the lowest abundance and diversity, with isolates in only four genera. For *P. quinquefolius*, culturing revealed that *Glomerella* species were the dominant endophytic fungi mostly in leaves and stems, while a *Cladosporium* sp. was mostly dominant in roots [45]. They observed a few examples of tissue specificity, such as *Colletotrichum higginsianum* and *Colletotrichum linicola* being only found in leaves. Averaged over the years, fungal endophyte diversity and abundance were highest over the years in roots, followed by leaves and lowest in stems. Both of those studies showed that roots had the highest fungal endophyte diversity and abundance, but differed in their results for other tissues. In contrast, ITS2 rRNA sequencing of *P. notoginseng* tissues showed that leaves had the highest diversity of fungi compared to roots and stems [26]. The Sordariomycetes and Glomeromycetes were abundant in roots, the Sympoventuriaceae in the Dothideomycetes were abundant in stems, and the Dothideomycetes, Diaporthales and Xylariales were abundant in leaves, all of which are Ascomycota. More studies are needed with sequence analysis of fungi in tissues of different *Panax* species to confirm those results. 

Fungal endophyte diversity and abundance can be affected by the age of ginseng plants. For 1- to 4-year-old *P. ginseng*, Park et al. [46] isolated 81 fungal endophytes and showed that the fewest species among nine fungal endophytes isolates was in 1-year-old roots while the most was in 3-year-old roots. A shift over time was also indicated by the dominant endophyte being *Fusarium solani* in 1-year-old roots, whereas *Phoma radicina* was dominant in 2-, 3- and 4-year-old roots. The beneficial fungus, *Trichoderma citrinoviride*, was only found in young roots, but several pathogens, such as *F. oxsporum* and *C. destructans*, were only found in older roots. For *P. notoginseng*, fungal diversity progressively increased in 1-, 2- and 3-year-old healthy roots (6 roots per age) measured using ITS1 sequences, and fungal endophytes in 1-year-old roots were almost entirely genera in the Ascomycota (98.5%) [44]. A notable change was higher abundance of the pathogen, *Cylindrocarpon*, in 3- versus 1- and 2-year-old roots. In contrast, the greatest diversity among 134 fungal endophyte isolates with *P. quinquefolius* was in 1-year-old roots progressively declining in -2, 3- and 4-year-old roots [45]. Populations appeared to shift over time, such as *Cladosporium* that was dominant in 1- to 3-year-old roots but not 4-year-old roots. A shift in populations over time was also indicated by the endophytic fungi of 4-year-old roots having the lowest similarity to those of the other ages. Most of these studies show that as ginseng roots become older, fungal endophyte species diversity and abundance increase. At least in part, that is due to increases in certain pathogens, even though the roots are symptomless. Perhaps such fungi are latent pathogens rather than truly endophytes. 

## 5. Ginseng Interactions with Fungal Endophytes

Compared to bacterial endophytes, there are fewer studies on the ability of fungal endophytes of ginseng to transform different types of ginsenosides. For example, an *Arthrinium* sp. isolated from *P. ginseng* roots used β-glucosidase to convert ginsenoside Rb1 to Rd then to F2 and finally the minor ginsenoside, compound K, which only has one sugar remaining [47]. However, some fungal endophytes of ginseng also appear to be able to directly produce different saponins in culture including ginsenosides. Among 38 isolates from roots of *P. ginseng*, *Fusarium, Aspergillus* and *Verticillium* isolates produced the highest concentration of triterpenoid saponins in potato dextrose broth, creating Rb2 or Rc [48]. The culture filtrates of those three fungal isolates differed, but all showed antimicrobial activity against two human bacterial pathogens as well as the ginseng fungal pathogen, *R. solani*. Among 53 fungal endophytes from roots and seeds of *P. notoginseng*, two were found to produce saponins in potato dextrose broth with an *Aspergillus* isolate synthesizing Re Rd, and 20(S)-Rg3 and a *Fusarium* isolate synthesizing Rb1, Rd and 20(S)-Rg3 [49]. The culture filtrates showed inhibitory activity against five human bacterial and three human yeast pathogens. If they also produced those antimicrobial compounds when growing inside plant tissues, then they could also potentially be used to control ginseng infections. 

In addition to saponins, other antimicrobial compounds are produced by fungal endophytes of ginseng that show disease biocontrol potential. Antifungal activity was observed among volatile organic compounds produced by the endophyte *Trichoderma gamsii* from *P. notoginseng* roots, which included dimethyl disulfide, dibenzofuran, methanethiol and ketones, that may act as fungal growth inhibitors [50]. Xu et al. [51] found the antifungal compound, falcarinol, from the endophytic fungus, a *Paecilomyces* sp., isolated from *P. ginseng*, and the fungus inhibited the fungal plant pathogen, *Pyricularia oryzae*, and four human pathogenic fungi. Xiong et al. [52] found six novel compounds, named myrothins, from a *Myrothecium* endophyte isolated from *P. notoginseng* seedlings, which showed activity against the root rot fungi, *F. oxysporum* and *Phoma herbarum*. Ethyl acetate extracts of cell free filtrates of 40 endophytes from different tissues of *P. ginseng* were tested against six different fungal pathogens of ginseng, and the extracts of *Colletotrichum pisi, F. oxysporum, F. solani, Phoma terrestris* and two unidentified fungi showed antimicrobial activity against one or more of six ginseng fungal pathogens, except for *C. destructans* [53]. Among the ethyl acetate extracts, the one from *P. terrestris* showed the strongest activity and contained the antifungal compound, 5H-dibenz [B, F] azepine, with lower amounts of the other antimicrobial compounds, 2-phenylindole, 2-butyl-5-ethylthiophene, dioctyl phthalate and hexadecanoic acid. While not tested on a ginseng disease, the extract reduced infection of Arabidopsis leaves by *B. cinerea.* Furthermore, the *P. terrestris* extract reduced the expression of four genes of *B. cinerea* involved in virulence or mycelial growth when added to a pure culture of the pathogen. Ethyl acetate extracts from 21 isolates of endophytic fungi from roots, leaves, stems, and seeds of *P. notoginseng* showed activity against one or more of five fungal root rot pathogens of ginseng with extracts from five endophytes having activity against all pathogens tested [44]. The endophyte extracts showing high activity against one or more of the pathogens were from *Cladosporium oxysporum, Pestalotiopsus uvicola, Colletotrichum gloeosporioides, Penicillium crustosum* and *Trichoderma koningiopsis*. However, none of the extracts were analyzed to identify the antifungal compounds. 

In addition to antimicrobial compounds, disease biocontrol can also be achieved by mycoparasitism and ISR in the host. An endophytic isolate of *T. gamsii* from *P. notoginseng* roots coiled around the hyphae of four plant pathogenic fungi in culture, typical for mycoparasitism by *Trichoderma* [50]. Soil application of the *T. gamsii* isolate also reduced root disease symptoms allowing greater seed emergence of *P. notoginseng* in a continuously cropped field as well as reduced root rot of older seedlings. An endophytic *T. citrinoviride* from mountain-cultivated *P. ginseng* exhibited mycoparasitism against six ginseng pathogens, penetrating or coiling around their hyphae [54]. The endophyte had endo-1,4-β-D-glucanase, exo-1,4-β-D-glucanase, β-1,3-glucanase and β-glucosidase activities that were induced when fungal cell walls were included as a substrate. Evidence that the isolate induced systematic resistance was that inoculating *P. ginseng* roots with *T. citrinoviride* spores prevented root rot by *C. destructans* and foliar infection by *B. cinerea*, as well as upregulated expression of ginseng genes involved in ginsenoside biosynthesis and defense genes in roots and/or stems and leaves. Field application of *T. citrinoviride* also showed decreased levels of foliar infection by *A. panax* associated with induced expression of several ginsenoside biosynthesis and defense genes. Inoculation of *P. ginseng* adventitious root cultures with immobilized spores of a *Chaetomium* endophyte of *P. ginseng* roots resulted in increased ginsenoside production and expression of several genes for ginsenoside synthesis as well as the defense genes, phenylalanine ammonia-lyase 2 and PR10–1 [55]. However, induced resistance against a pathogen was not examined. 

## 6. Ginseng Archaeal Endophytes

Evidence thus far for archaeal endophytes in ginseng comes from a metagenomics study of DNA extracted from healthy disinfected root tissue, where 5% of the reads matched archaeal sequences, but no further description was provided [30]. While much lower than the 81% of reads matching bacteria, it was not that much lower than the 12% of reads matching eukaryotes, presumably mostly fungi. Thus, archaea are rare, but not extremely rare, inside ginseng. In other plants, archaeal endophytes have been identified primarily belonging to the phyla Crenarchaeota, Thaumarchaetoa and Euryarchaeota [56], and this is likely similar in ginseng. Due to the unique properties of archaea that allow them to survive in an extremely diverse range of environments, it is possible that they could provide stress tolerance metabolites for the plants that they inhabit [57]. At this point, the study of archaeal endophytes has been primarily limited to detection in plants. However, the effect of environmental conditions on their abundance and diversity was examined in maize roots which contained more archaea when plants were grown with organic fertilizer than inorganic or no fertilizer, with 15 genera of archaea more abundant in roots with organic fertilizer [58]. The limited research on archaeal endophytes in plants is indicated by Fadiji et al. [58], who reported that 10 genera of archaea detected in maize roots in their study had never been previously described as endophytes in maize. Much more research is needed on archaeal endophytes of ginseng.

## 7. Ginseng Viral Endophytes

Reports of cryptic/persistent viruses in ginseng species is limited to Hong et al. [19], who reported that 2% of the reads in a metagenomics analysis of healthy *P. ginseng* roots matched viral sequences. However, no further description was given. In other plants, examples of cryptic/persistent viruses include ArCV-1, a tripartite ds RNA virus in the *Deltpartitivirus* found in *Cajanus cajan* [59], RCV-1, a tripartite dsRNA in the *Partitivirius* identified in *Rosa multiflora* [60] and FCIL, a tripartite dsRNA in the Partitiviridae, identified in *Fragaria chiloensis* [61]. It may be that viruses endophytic in *Panax* species would be similar. It is even possible that endophytic viruses could benefit their host. Inoculation of brome mosaic virus, cucumber mosaic virus, tobacco mosaic virus and tobacco rattle virus onto ten monocot and dicot plants delayed drought symptoms, and cucumber mosaic virus infection of beet plants also showed increased frost tolerance [62]. The benefits was proposed to be due to increased levels of several osmoprotectants and antioxidants in virus-infected plants, thus demonstrating that plant viral infection can improve the tolerance of plants to abiotic stresses. However, it is unknown if cryptic/persistent virus infections would provide plants with the same benefits. 

## 8. Conclusions

*Panax* species contain a wide range of endophytes, similar to other plants. Among the 16 *Panax* species, studies have almost exclusively been limited to *P. quinquefolius, P. ginseng* and *P. notoginseng*. Thus, comparison between a wider range of ginseng species would be desirable, particularly to determine if the different geographical distributions or types of environments of the different *Panax* species have impacts on their endosphere microbiomes. The widespread occurrence of endosphere microbes with the ability to metabolize ginsenosides or produce ginsenosides in culture indicates that although the endophyte species found in ginseng are not unique, their genomes may differ from isolates of the same species in other environments, having altered or unique genes to help them grow in the specialized conditions of the ginseng apoplast. Genomic sequencing of ginseng endophytes would be useful to compare the genomes of isolates of ginseng endophytes to those of the same species found in environments such as soil, water, other plants or even animals. This could even be applied to compare the genomes of endophyte isolates from different tissues of the same ginseng plant, the same ginseng species from cultivated versus wild plants from different locations and even different species of ginseng. 

While diversity and abundance of endophytes have been compared between tissues, environments and plant ages, much less work has been performed examining the benefits of endophytes for plant growth promotion, particularly fungal endophytes. Considering the ability of fungal endophytes in promoting growth of other plant species, such as by producing IAA, ammonia and solubilizing phosphate [63], it would be expected that similar benefits could be provided by fungal endophytes of ginseng. Both bacterial and fungal endophytes have been examined for improving resistance to plant diseases, but most of that work has concentrated on direct antimicrobial effects in culture against plant pathogens with much fewer studies testing them for mycoparasitism or induced disease resistance. Additionally, there have been few examples of their use in field trials to control disease. Many bacterial and fungal endophytes of other crops have been shown to reduce abiotic stress [8,64], but this has yet to be examined in ginseng. This could greatly improve ginseng production as stresses from many factors, such as unfavorable nutrient, water and temperature conditions, can occur [65]. Additionally, there are many concerns about the degradation of soil physiochemistry during the growth of ginseng, which may be related to the common failure of ginseng to grow in soils previously used for ginseng cultivation, known as ginseng replant disease or replant disorder [65,66]. The use of microbial inoculants containing rhizosphere bacteria has been reported to reduce replant disease [67], and using endosphere microbes may be similarly or even more effective. This review has shown that there is a diverse range of endophytes in all types of ginseng tissues, and some of those endophytes have the potential to provide a more environmentally friendly approach to improving ginseng production in the field. Much more research on ginseng endophytes is needed to realize their potential.

## Data Availability

Not applicable.
